# Effect of Autophagy Modulators on Vascular, Glial, and Neuronal Alterations in the Oxygen-Induced Retinopathy Mouse Model

**DOI:** 10.3389/fncel.2019.00279

**Published:** 2019-06-26

**Authors:** Paula V. Subirada, María C. Paz, Magali E. Ridano, Valeria E. Lorenc, Claudio M. Fader, Gustavo A. Chiabrando, María C. Sánchez

**Affiliations:** ^1^Departamento de Bioquímica Clínica, Facultad de Ciencias Químicas, Universidad Nacional de Córdoba, Córdoba, Argentina; ^2^Consejo Nacional de Investigaciones Científicas y Técnicas (CONICET), Centro de Investigaciones en Bioquímica Clínica e Inmunología (CIBICI), Córdoba, Argentina; ^3^Nanomedicine and Vision Group, Facultad de Ciencias Biomédicas, Instituto de Investigaciones en Medicina Traslacional, Universidad Austral, Consejo Nacional de Investigaciones en Ciencia y Tecnología (CONICET), Pilar, Argentina; ^4^Facultad de Odontología Mendoza, Universidad Nacional de Cuyo, Mendoza, Argentina; ^5^Instituto de Histología y Embriología (IHEM), Facultad de Ciencias Médicas, Universidad Nacional de Cuyo, Consejo Nacional de Investigaciones Científicas y Técnicas (CONICET), Mendoza, Argentina

**Keywords:** autophagy, hypoxia, proliferative retinopathies, vascular endothelial growth factor, retinal functionality, gliosis, neurodegeneration

## Abstract

Hypoxia is one of the main insults in proliferative retinopathies, leading to neovascularization and neurodegeneration. To maintain homeostasis, neurons require efficient degradation and recycling systems. Autophagy participates in retinal cell death, but it is also a cell survival mechanism. Here, we analyzed the role of autophagy at the three characteristic time periods in the oxygen-induced retinopathy (OIR) mouse model and determined if its modulation can improve vascular and non-vascular alterations. Experiments were performed with chloroquine (CQ) in order to monitor autophagosome accumulation by lysosomal blockade. Post natal day (P)17 OIR mouse retinas showed a significant increase in autophagy flux. In particular, an intense LC3B and p62 staining was observed in inner layers of the retina, mainly proliferating endothelial cells. After a single intraocular injection of Rapamycin at P12 OIR, a decreased neovascular area and vascular endothelial growth factor (VEGF) protein expression were observed at P17 OIR. In addition, whereas the increased expression of glial fibrillary acidic protein (GFAP) was reversed at P26 OIR, the functional alterations persisted. Using a similar therapeutic schedule, we analyzed the effect of anti-VEGF therapy on autophagy flux. Like Rapamycin, VEGF inhibitor treatment not only reduced the amount of neovascular tufts, but also activated autophagy flux at P17 OIR, mainly in ganglion cell layer and inner nuclear layer. Finally, the effects of the disruption of autophagy by Spautin-1, were evaluated at vascular, glial, and neuronal levels. After a single dose of Spautin-1, Western blot analysis showed a significant decrease in LC3B II and p62 protein expression at P13 OIR, returning both autophagy markers to OIR control levels at P17. In addition, neither gliosis nor functional alterations were attenuated. In line with these results, TUNEL staining showed a slight increase in the number of positive cells in the outer nuclear layer at P17 OIR. Overall, our results demonstrate that all treatments of induction or inhibition of the autophagic flux reduced neovascular area but were unable to completely reverse the neuronal damage. Besides, compared to current treatments, rapamycin provides a more promising therapeutic strategy as it reduces both neovascular tufts and persistent gliosis.

## Introduction

Retinal neovascular pathologies are still leading causes of blindness worldwide in middle age (DR) and pediatric [retinopathy of prematurity (ROP)] population (Campochiaro, 2015; Rubio and Adamis, 2016). Currently, there is a wide spectrum of treatments available for these diseases including laser photocoagulation and, more recently the intraocular injections of anti-VEGF agents (Evans et al., 2014; Diabetic Retinopathy Clinical Research Network et al., 2015; Tah et al., 2015). Independently of the etiology, retinal neovascular pathologies are characterized by an increase in cytoplasmic HIF-1α levels, a subsequent dimerization with HIF-1β and translocation to the nucleus (Semenza, 2012). Vascular endothelial growth factor is one of the most critical target genes of HIF, and a well-known key player in neovascularization (NV) (Nagy et al., 2008). This trophic factor has pleiotropic functions over neurons and ECs in health and disease (Saint-Geniez et al., 2008; Zhang et al., 2009). In hypoxic environment VEGF levels largely increase and is the main responsible of ECs survival, migration, and proliferation (Gerhardt, 2008). Nowadays, anti-VEGF therapies are the most recommended treatment for retinal NV as they have proved high efficacy in animal models and clinical trials (Tah et al., 2015). Although anti-VEGF treatment has shown better outcomes than alternative treatments, ophthalmologists have detected unequal response of patients to the same administration scheme. Moreover, they have observed that some patients lost visual acuity after the chronic treatment (Osaadon et al., 2014; Yang et al., 2016). There are two main explanations that shed light on these events. On one hand, other proteins involved in NV, inflammation, vascular tone and metabolism have critical roles, whereby, VEGF inhibition would result insufficient to restore retinal homeostasis and regresses NV in the hypoxic milieu. On the other hand, VEGF is also a neuroprotective factor and its further depletion has deleterious effects over neuronal survival.

Consequently, many researchers have designed new pharmacological strategies to cope with NV through the modulation of different proteins or molecular pathways. Based on multiple studies carried out in the field of cancer angiogenesis, recently, it has been proposed that autophagy flux would have an important role as a potential therapeutic tool in clinical medicine (Mizushima and Komatsu, 2011). Autophagy is a catabolic intracellular mechanism that provides recycling of its components through the engulfment of damaged proteins and organelles in a double membrane vesicle (Levine et al., 2015). As it participates in retinal cell death but it is also a cell survival mechanism, its modulation may be either beneficial or deleterious depending on the retinal cell type involved or the disease context (Boya et al., 2016; Chai et al., 2016; Rosa et al., 2016; Amato et al., 2018). In this work, we intend to unravel the role of autophagy on retinal pathological NV and neurodegeneration in mice with oxygen-induced retinopathy (OIR), an animal model of proliferative ischemic retinopathy, and determine if its modulation can improve retinal functionality, gliosis, and avoid neuronal cell death.

## Materials and Methods

C57BL/6J mice were handled according to guidelines of the ARVO Statement for the Use of Animals in Ophthalmic and Vision Research. Experimental procedures were designed and approved by the Institutional Animal Care and Use Committee (CICUAL) of the Faculty of Chemical Sciences, National University of Córdoba (Res. HCD 1216/18). All efforts were made to reduce the number of animals used.

### Oxygen-Induced Retinopathy (OIR) Mouse Model

In the OIR mouse model (Smith et al., 1994), litters of mice pups and their mothers were exposed to high oxygen concentration (75 ± 2%) from P7 to P12 (hyperoxic period) in an incubator chamber. Oxygen was checked twice daily with an oxygen analyzer (Teledyne Analytical Instruments, Industry, CA, United States). Then, mice were brought to room air (RA) for additional 5 or 14 days. Age-matched control C57BL/6 mice were kept continuously at RA. Animals were maintained in clear plastic cages with standard light cycles (12 h light/12 h dark). At P12, OIR mice were intravitreally injected with a 1.0 μl of solution containing a specific treatment: (a) Rapamycin: 0.5 μg/ml (R0395 Sigma Aldrich, St. Louis, MO, United States); (b) Anti-VEGF mAb: 1.25 μg anti-VEGF diluted 1/20 with phosphate buffered saline (PBS) (Bevacizumab; Genentech, San Francisco, CA, United States) as previously reported (Ridano et al., 2017); (c) Spautin-1 200 μM (SML0440, Sigma Aldrich, St. Louis, MO, United States). Vehicle (DMSO/PBS or PBS)/-injected mice were used as controls. Rapamycin and Spautin-1 concentrations were selected according to *in vitro* assays (Chen et al., 2014; Russo et al., 2018) and concentrations 10 folds higher than in cell cultures were used (taking in account the dilution of the drug in the vitreous cavity). Briefly, pups were locally anesthetized with one drop of proparacaine hydrochloride 0.5% (Anestalcon, Alcon), exophthalmia was induced with one drop of tropicamide 1% (Midril, Alcon, Buenos Aires, Argentina) and eyes were injected at the upper nasal limbus as described previously (Barcelona et al., 2011). Some mice were sacrificed at three typical times in the OIR mouse model: P12 (maximum vaso-obliteration, VO), P17 (maximum NV) and P26 (vascular alteration resolution) (Smith et al., 1994). To evaluate the effect of Rapamycin or Spautin-1 mice were sacrificed 24 h after the injection (P13). In order to analyze the autophagy flux, some mice received an intraperitoneal (i.p.) injection of chloroquine (CQ) 60 mg/kg (Sigma Aldrich, St. Louis, MO, United States) diluted in sterile PBS 4 h before sacrifice. Eyes or retinas of sacrificed mice with CQ pre-treatment were collected and processed for Western blot and immunofluorescence assays and without CQ pre-treatment for quantitative real-time PCR (qRT-PCR), immunohistochemistry, or flat-mount assays. At least six mice per group were used for each condition at each survival time examined. Data were collected from both males and females as there were no apparent sex differences. All mice were sacrificed at the same time of day in order to avoid the circadian effects and to reduce the mouse-to-mouse variability in autophagy markers and flux. Lysosomal analyses were carried out with intraocular injection of 1 μl of red DQ-BSA (1 mg/ml dissolved in PBS pH 7.2; D12051, Invitrogen). The reagent is taken up by every cell and then the red dye is detected in acidic compartments, where DQ is hydrolyzed from albumin. Mice were sacrificed 4 h after DQ injection (without CQ pre-treatment), fixed with 4% paraformaldehyde (PFA) and dehydrated by sucrose gradient for further cryosection.

### Electroretinography (ERG)

Electroretinographic activity was assessed as previously described (Ridano et al., 2017). Briefly, after overnight (ON) dark adaptation and under dim red illumination, mice were anesthetized via i.p. injections with a solution containing ketamine (90 mg/kg)/xilacine (8 mg/kg). The pupils were dilated with 1% tropicamide and the cornea was lubricated with gel drops of 0.4% polyethyleneglycol 400 and 0.3% propylene glycol (Systane, Alcon, Buenos Aires, Argentina) to prevent damage. Mice were exposed to stimuli at a distance of 20 cm. A reference electrode was inserted on the back in the neck, a grounding electrode was attached to the tail, and a gold electrode was placed in contact with the central cornea. Electroretinograms were simultaneously recorded from both eyes and 10 responses to flashes of unattenuated white light (5 cd.s/m^2^, 0.2 Hz) from a photic stimulator (light-emitting diodes) set at maximum brightness were amplified, filtered (1.5-Hz low-pass filter, 1000 high-pass filter, notch activated) and averaged (Akonic BIO-PC, Argentina). The a-wave was measured as the difference in amplitude between the recording at onset and trough of the negative deflection, and the b-wave amplitude was measured from the trough of the a-wave to the peak of the b-wave. The latencies of the a- and b-waves were measured from the time of flash presentation to the trough of the a-wave or the peak of the b-wave, respectively. Responses were averaged across the two eyes for each mouse.

### Labeling of Flat-Mount Retinas

Mice were euthanized at P17 and eyes were enucleated and fixed with freshly prepared 4% PFA for 1–2 h at room temperature (RT). Corneas were removed with scissors along the limbus and the whole retinas were dissected. Then, they were blocked and permeabilized in Tris-buffered saline (TBS) containing 5% Bovine Serum Albumin (BSA) and 0.1% Triton-X-100 during6 h at 4°C. After that, retinas were incubated ON with 0.01 μg/μl of Isolectin IB4 Alexa fluor-488 conjugate (GSA-IB4) from Molecular Probes, Inc. (Eugene, OR, United States) and anti-GFAP (1/200; Dako, Carpinteria, CA, United States) or anti- microtubule-associated protein-1 light-chain 3 (LC3) B (1:100; L7543, Sigma). Retinas were then washed with TBS containing 0.1% Triton-X-100, stored in PBS at 4°C and examined by confocal laser-scanning microscopy (Olympus FluoView FV1200; Olympus, Corp., New York, NY, United States).

### Retinal Cryosection, Protein Extract, and RNA Sample Preparation

For cryosection, enucleated eyes were fixed during 2 h with 4% PFA at RT, and incubated ON in 10, 20, and 30% of sucrose in PBS at 4°C. Then, they were embedded in optimum cutting temperature (OCT) (Tissue-TEK, Sakura) compound, and 10 μm-thick radial sections were obtained by using a cryostat, as described (Sanchez et al., 2006). Retinal cryosections were stored at -20°C under dry conditions until immunohistochemical analysis.

Neural retinas were dissected from RPE/choroid layers for Western blot and qRT-PCR analysis. Protein extracts were obtained from retinas after homogenization with a lysis buffer containing 20 mM Tris-HCl pH 7.5, 137 mM NaCl, 2 mM EDTA pH 8, 1% Nonidet P40, 1 mM phenylmethylsulfonyl fluoride (PMSF), 2 mM sodium ortovanadate and protease inhibitor cocktail (Sigma Aldrich, St. Louis, MO, United States) (Sanchez et al., 2006), and were sonicated during 20 s at 40% amplitude. In addition, some neural retinas were disrupted in 500 μL Trizol (Invitrogen) and were stored at -80°C until RNA extraction. All the assays were performed in triplicate and results are representative of at least three independent experiments (animals in each group).

### Immunofluorescence

Immunostaining was performed as described previously (Ridano et al., 2017). Briefly, mouse cryosections were washed in PBS, blocked with 2% of BSA in PBS containing 0.1% Tween-20, for 1 h and then incubated ON at 4°C with the following primary antibodies, respectively: rabbit polyclonal anti-LC3B (1/100; L7543, Sigma Aldrich), mouse monoclonal anti-p62 (1/100; ab56416, Abcam), rabbit polyclonal anti- GS (1/100; ab16802, Abcam), rat monoclonal anti- LAMP1 (1/350; ab25245, Abcam), mouse monoclonal anti- GS (1/100; MAB 302, Millipore), mouse monoclonal anti-CD31 (1/50; Abcam, Inc., Cambridge, MA, United States) and rabbit polyclonal anti-NG-2 (1/100; AB5320, Millipore). Then, sections were washed with TBS 0.1% Triton-X-100 and incubated with secondary antibodies including goat against rabbit or mouse IgG conjugated with Alexa Fluor 488 and 594 (1/250; Molecular Probes, Eugene, OR, United States) and goat anti rat IgG conjugated with Alexa Fluor 594 (1/200; ab150160, Abcam), during 1 h at RT. The sections were also counterstained with Hoechst 33258 (1:3000; Molecular Probes) for 7 min. After a thorough rinse, the sections were mounted with Fluor Save (Calbiochem, La Jolla, CA, United States) and cover slipped. The labeling was visualized using a confocal laser-scanning microscope (Olympus Fluvial FV300 or FV1200; Olympus, Corp., New York, NY, United States). Finally, images were processed with Image J 2 software (National Institutes of Health, Bethesda, MD, United States), including spatial deconvolution, vesicle quantification and colocalization. Negative controls without incubation with primary antibody were carried out to detect unspecific staining (data not shown).

### Western Blot

Protein concentration of retinal extracts were determined by a BCA kit (Pierce, Buenos Aires, Argentina) and 10–20 μg of proteins were electrophoresed in 15% SDS-PAGE. After electrophoresis, proteins were transferred to nitrocellulose membranes (Amersham Hybond ECL; GE Healthcare Bio-Sciences AB, Uppsala, Sweden). To prevent non-specific binding, membranes were blocked with 5% milk in TBS containing 0.1% Tween-20 (TBST) during at least 1 h at RT. Then, blots were incubated with primary antibodies diluted in TBST or 5% BSA in TBST for 1 h at RT or overnight at 4°C, according to the antibody. The following antibodies were used: rabbit polyclonal anti-LC3 (1/1000; L7543, Sigma Aldrich), mouse monoclonal anti p62 (1/1000; ab56416, Abcam) mouse monoclonal anti- VEGF (1/500; R&D system), rabbit polyclonal anti-GFAP (1/1000; Dako, Carpinteria, CA, United States), mouse monoclonal anti-GS (1/500; MAB 302, Millipore Corporation MA, United States), rabbit polyclonal anti-caspase 3 (1/300; HPA 002643, Sigma Aldrich) and mouse monoclonal anti-β-actin (1/2000; ab8226, Abcam). Blots were incubated with IRDye 800 CW donkey anti-rabbit Ig, or IRDye 800 CW donkey anti-mouse IgG antibodies (1/15000 in TBS with 5% BSA) for 1 h, protected from light. After washing with TBST, membranes were visualized and quantified using the Odyssey Infrared Imaging System (LI-COR, Inc., Lincoln, NE, United States).

### Quantitative Real-Time Polymerase Chain Reaction

Total RNA was extracted from neural retinas using Trizol (Invitrogen), according to the manufacturer’s instructions and was processed as previously reported (Ridano et al., 2017). Briefly, 1 μg of total RNA was reverse-transcribed in a total volume of 20 μl using random primers (Invitrogen, Buenos Aires, Argentina) and 50 U of M-MLV reverse transcriptase (Promega, Corp.). Then, cDNA was mixed with 1x SYBR Green PCR Master Mix (Applied Biosystems) and forward and reverse primers: Beclin1 forward: ATGGAGGGGTCTAAGGCGTC/ Beclin 1 reverse: TGGGCTGTGGTAAGTAATGGA; ATG5 forward: TGTGCTTCGAGATGTGTGGTT/ ATG5 reverse: GTCAAATAGCTGACTCTTGGCAA; MAPLC3 forward: CGCTTGCAGCTCAATGCTAAC/ MAPLC3 reverse: TCGTACACTTCGGAGATGGG; P62 forward: TGTGGAACATGGAGGGAAGAG/ P62 reverse: TGTGCCTGTGCTGGAACTTTC. qPCR were carried out on an Applied Biosystems 7500 Real-Time PCR System with Sequence Detection Software v1.4. The cycling conditions included a hot start at 95°C for 10 min, followed by 40 cycles at 95°C for 15 s and 60°C for 1 min. Specificity was verified by melting curve analysis. Results were normalized to β-actin (Forward: GGCTGTATTCCCCTCCATCG/ Reverse: CCAGTTGGTAACAATGCCATGT). Relative gene expression was calculated according to the 2-ΔΔCt method. Each sample was analyzed in triplicate. No amplification was observed in PCRs using as template water during the cDNA synthesis (data not shown).

### TUNEL Assay

Cell death was examined by terminal deoxynucleotidyltransferase biotin dUTP nick end labeling (TUNEL) assay (Roche, Mannheim, Germany), which contain an anti-dUTP antibody labeled with peroxidase (POD), according to manufacturer’s instructions. Slides were counterstained with methyl green to visualize total nuclei and then mounted with DPX Mounting Media (Sigma Aldrich, St. Louis, MO, United States). Negative controls without enzyme were processed in order to avoid false positive results (data not shown). Images were obtained under a light microscope (Nikon Eclipse TE2000-E, United States).

### Statistical Analysis

Statistical analysis was performed using the GraphPad Prism 5.0 software. A *p*-value < 0.05 was considered statistically significant. Parametric or non-parametric tests were used according to variance homogeneity evaluated by F or Barlett’s tests. Two-tailed unpaired t or Mann–Whitney tests were used in analysis of two groups. One-way analysis of variance (ANOVA) followed by Dunnett´s multiple comparison post-test or Kruskal–Wallis followed by Dunn’s multiple comparison post-test were used to determine statistical significance among more than two different groups. Two-way ANOVA followed by Bonferroni post-test was used in comparisons between groups when two variables were affecting the dependent variable. Mean ± standard error (SEM) are shown in graphs analyzed with parametric tests and median with interquartile range are shown when data were analyzed with non-parametric test.

## Results

### Hypoxia Induced Autophagy During Neovascular Stage in the OIR Mouse Model

**FIGURE 1:**
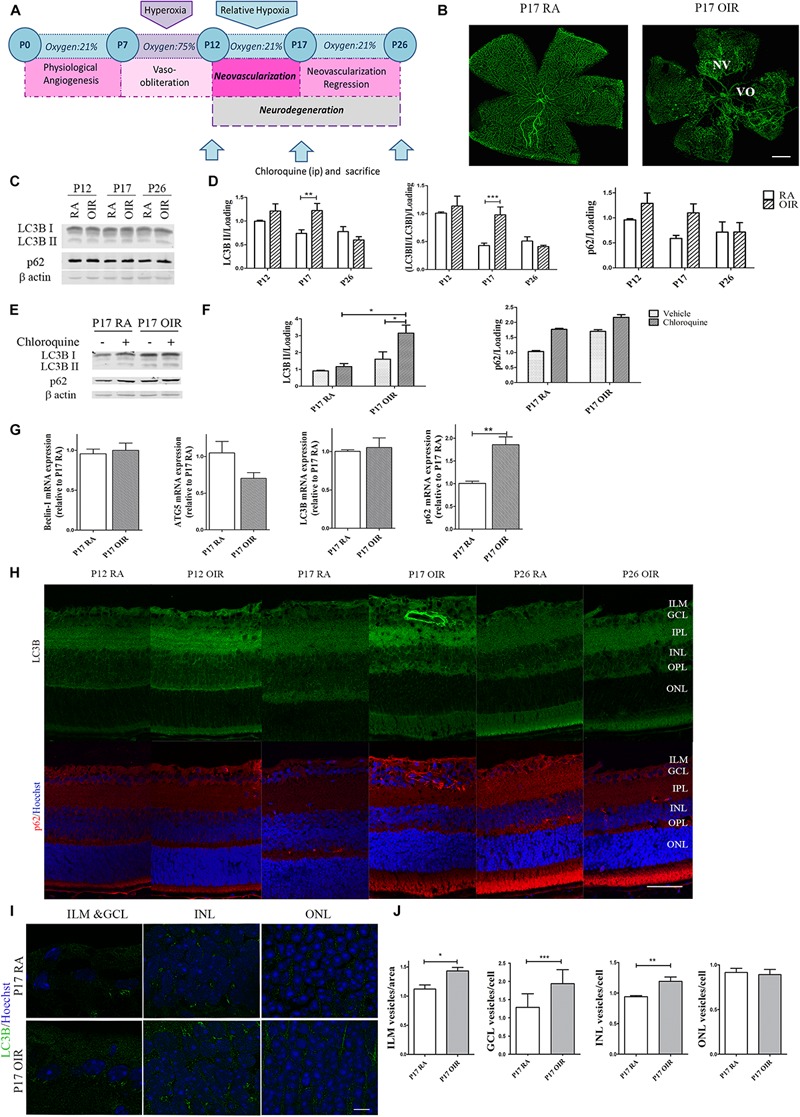
Effect of hypoxia on the autophagic flux during NV and neurodegeneration in OIR mouse retinas. **(A)** Scheme representing the OIR mouse model with hallmark time points during experimental disease development. Neonatal mice and their nursing mother are kept in room air from birth to P7. At P7, they are exposed to 75% oxygen, which inhibits retinal vessel growth and causes significant VO. Mice are returned to RA at P12; the avascular retina becomes hypoxic, eliciting both normal vessel regrowth and pathological neovascular response. NV reaches its maximum at P17. Shortly thereafter, it spontaneously regresses and the vascular alterations resolve by P25. **(B)** Representative images of flat-mount retinas at P17 showing GSA-IB4 vascular staining in RA and OIR mice. Areas with VO and NV are indicated. Scale bar: 500 μm. **(C,D)** Representative Western blot and quantification data of autophagy markers LC3B II and p62 from neural retinal extracts of RA and OIR at P12, P17, and P26. Mice were injected i.p. with CQ 4 h before sacrifice. β-Actin is shown as a loading control. Graph shows results of three independent experiments. **(E,F)** Representative Western blot and quantification data of autophagy markers, LC3B II and p62, from neural retinal samples of RA and OIR mice injected with vehicle or CQ at P17, and evaluated 4 h after administration. β-Actin is shown as a loading control. Graph shows results of three independent experiments. **(G)** Beclin-1, ATG5, LC3, and p62 mRNA levels were quantified by qRT-PCR in neurosensory retinas of P17 OIR and RA mice. Results were normalized to β-actin and expressed according to the 2-ΔΔCt method using as calibrator the mRNA level obtained from P17 RA mouse retinas. **(H)** Representative immunofluorescence analysis of LC3B (green) and p62 (red) in cryosections of RA and OIR mice injected with CQ at P12, P17, or P26 and evaluated 4 h after administration. Cell nuclei were counterstained with Hoechst 33258 (blue). **(I)** Representative labeling of LC3B (green) in cryosections of RA and OIR mouse retinas at P17, 4 h after CQ administration. Images were taken with oil 60X objective in the best confocal resolution condition. Cell nuclei were counterstained with Hoechst 33258 (blue) Scale bar: 5 μm. **(J)** Quantification of LC3B puncta per area or cell with ImageJ analyze particles software. ILM, inner limiting membrane; GCL, ganglion cell layer; IPL, inner plexiform layer; INL, inner nuclear layer; OPL, outer plexiform layer; ONL, outer nuclear layer. Data are presented as mean ± SEM. ^∗^*p* < 0.05, ^∗∗^*p* < 0.01, ^∗∗∗^*p* < 0.001.

Autophagy is a strictly regulated process that plays a vital role in cell growth, transition, and death (Kimura et al., 2017). In the nervous system it is a matter of intense investigation as these pathways are often missregulated during neovascular and neurodegenerative conditions (Harris and Rubinsztein, 2011; Du et al., 2012; Liu et al., 2016). In this regard, the mouse model of OIR offers an opportunity to examine the role of hypoxia in the pathogenesis of retinal NV, neuroinflammation, oxidative stress, and neurovascular cross-talk (Kim et al., 2016). Thus, in order to study autophagy flux, we determined the expression of classical autophagy markers at the three relevant time points in the OIR mouse model (Figure 1A). As we expected, GSA-IB4 lectin-labeled blood vessels in flat-mount retinas showed, at P17 OIR, a central zone of VO in addition to the characteristic vitreoretinal neovascular tufts, which were not observed in P17 RA controls (Figure 1B). Post-mitotic cells, especially neurons, are highly dependent on intracellular degradation systems because they provide adequate waste products elimination. The retina is known to have a fast autophagy flux due to the constant exposure to light and increased production of reactive oxygen species (Trachsel-Moncho et al., 2018). Therefore, we decided to block the autophagosome fusion with lysosomes and degradation with an i.p. injection of CQ to evaluate the accumulation of autophagosomes. Retinal levels for LC3B and p62 protein expression, at each temporal point, were measured in both RA and OIR mice by Western blot assays (Figure 1C). Quantitative analysis revealed a slight increase in LC3B II protein expression level at P12 in OIR mice, which was markedly upregulated in P17, returning to baseline levels at P26. In our experimental conditions, upregulation of LC3B II in retinal extracts at P17 OIR was associated with an increase, although not statistically significant, in p62 protein expression (Figure 1D). To gain more insight into autophagy regulation at the time point where most changes have been observed (P17), additional Western blot assays in mice injected with vehicle or CQ were performed (Figure 1E). Quantitative analysis showed an increase, although not statistically significant, in both LC3B II and p62 levels at P17 OIR compared to RA mice retinas injected with vehicle, which were further enhanced after CQ treatment (Figure 1F). Our data showed that the augmentation of LC3B II by hypoxic injury is due to the enhancement of autophagic flux rather than impaired clearance of autophagosomes.

Next, we examined the transcript expression of molecules involved in nucleation (Beclin-1), elongation (ATG-5), and structure (LC3) of the autophagosome (Yin et al., 2016), and also the autophagy substrate p62 at the peak of NV. Beclin-1, ATG-5 and LC3 mRNA levels did not showed changes at P17, whereas expression of p62 mRNA was increased ∼1.8-fold at this time point (Figure 1G).

By immunostaining of retinal cryosections we confirmed the increase in LC3B and p62 protein expression at P17 OIR (Figure 1H). Given that there is a tissue gradient of hypoxia in OIR retinas (Rodrigues et al., 2013), we decided to evaluate whether variations in autophagy flux were similar in every retinal layer. The analysis revealed an intense LC3B and p62 signal in the inner limiting membrane (ILM) at P17 OIR (Figure 1I). As neurons in the INL and GCL are severely affected by hypoxia (Ridano et al., 2017), quantification evidenced an increase in LC3B puncta mainly in these areas. At the same time, no modification in the autophagy marker puncta was observed in the ONL (Figure 1I,J).

**FIGURE 2:**
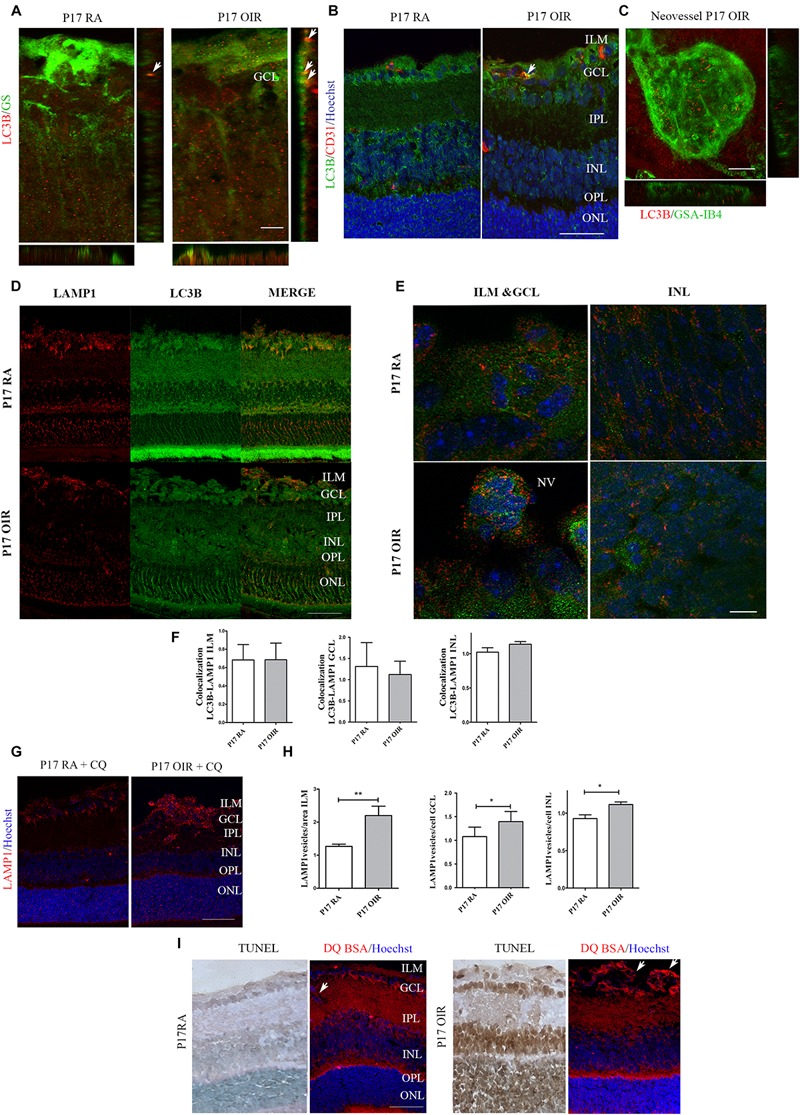
Effect of hypoxia on LC3B expression and distribution in P17 mouse retinas. Double labeling using: **(A)** a mouse monoclonal antibody for LC3B (red) and a MGC marker, anti-GS (green). Representative merged fluorescence confocal images of entire z-stack (XY) with orthogonal views, XZ and YZ of a mouse retina. Arrows are indicating autophagosomes in MGCs. Scale bar: 5 μm; **(B)** a mouse monoclonal antibody for LC3B (green) and an EC-specific marker, anti-CD31 (red). Cell nuclei were counterstained with Hoechst 33258 (blue). Arrows are indicating autophagosomes in ECs. Scale bar: 50 μm. **(C)** High magnification confocal micrograph of the representative P17 OIR retina showing a typical neovascular tuft (GSA-IB4, green) staining with LC3B (red). Images were taken with oil 60X objective in the best confocal resolution condition. Representative merged fluorescence confocal images of entire z-stack (XY) with orthogonal views, XZ and YZ. Scale bar: 15 μm **(D)** Representative immunofluorescence analysis of LAMP1 (red) and LC3B (green) in cryosections of P17 RA and OIR mice. Scale bar: 50 μm. **(E)** Representative immunofluorescence analysis of LAMP1 (red) and LC3B (green) in cryosections of P17 RA and OIR mice. Images were taken with oil 60X objective and zoom 5x in the best confocal resolution condition. Cell nuclei were counterstained with Hoechst 33258 (blue) Scale bar: 5 μm. **(F)** Quantification of LC3B/LAMP1 vesicles per area (ILM) or cell (GCL and INL) with ImageJ JACoP software. Pearson values were compared statistically. Statistical *t*-test was performed. **(G)** Representative immunofluorescence analysis of LAMP1 (red) in cryosections of RA and OIR mice injected with CQ at P17, and evaluated 4 h after administration. Scale bar: 50 μm. **(H)** Quantification of LAMP1 puncta per area or cell with ImageJ analyze particles software. Data are presented as mean ± SEM. ^∗^*p* < 0.05, ^∗∗^*p* < 0.01. **(I)** Representative TUNEL-labeled cryosections of P17 RA and OIR mice and cryosections of mice intravitreally injected with bovine serum albumin derivate DQ-BSA and co stained with anti-LC3B (green). Arrows are indicating DQ-BSA staining in ECs. Scale bar: 50 μm. ILM, inner limiting membrane; GCL, ganglion cell layer; IPL, inner plexiform layer; INL, inner nuclear layer; OPL, outer plexiform layer; ONL, outer nuclear layer; NV, neovascularization.

Finally, we explored in more detail the presence of autophagosomes in the ILM, a region where vascular and macroglial cells actively interact. In cryosections, we identified Müller glial cells (MGCs) and vascular cells by staining with GS and CD31 respectively, whereas in retinal flat-mounts ECs were isolectin IB4^+^ cells (Figure 2, upper panel). Both macroglial and ECs showed, at P17 OIR, a slight increase in LC3B vesicles within the cells (Figure 2A,B). However, in neovascular tufts, ECs showed the great number of autophagic vesicles (Figure 2C).

In order to determine if the degradative pathway is activated at P17 OIR, we performed immunofluorescence assays using a combination of LC3B antibody along with the late endosomal compartments and lysosomes marker LAMP1 antibody (Figure 2D). Fusion of LC3B and LAMP1 positive vesicles was observed in ILM, GCL, and INL in OIR and RA neural retinas (Figure 2E). Our statistical analysis did not found differences in colocalization quantification in absence of CQ, probably due to the fast flux in the retina (Figure 2F). Through the injection of CQ, we could detect an increase of LAMP-1 vesicles, suggesting that lysosomes and late endosomes are following the degradation pathway properly (Figure 2G,H). To further examine the lysosomal activity, mice were intravitreally injected with bovine serum albumin derivate DQ-BSA (Frost et al., 2017). Confocal images showed an increased DQ-BSA staining in ECs at the time when the NV peaks in the OIR mice, compared to RA control retinas (Figure 2I), correlating with the observed in Figure 2E. However, this increase was not observed in the retinal layers where death was increased (Figure 2I). Collectively, our results showed an increase in autophagy flux mainly in proliferating ECs, and inner layers of the retina whereas neurons from the ONL did not showed modifications in autophagic flux at P17 OIR.

### Rapamycin Promoted Autophagy, Prevented NV and Improved Gliosis in the OIR Mice Model

**FIGURE 3:**
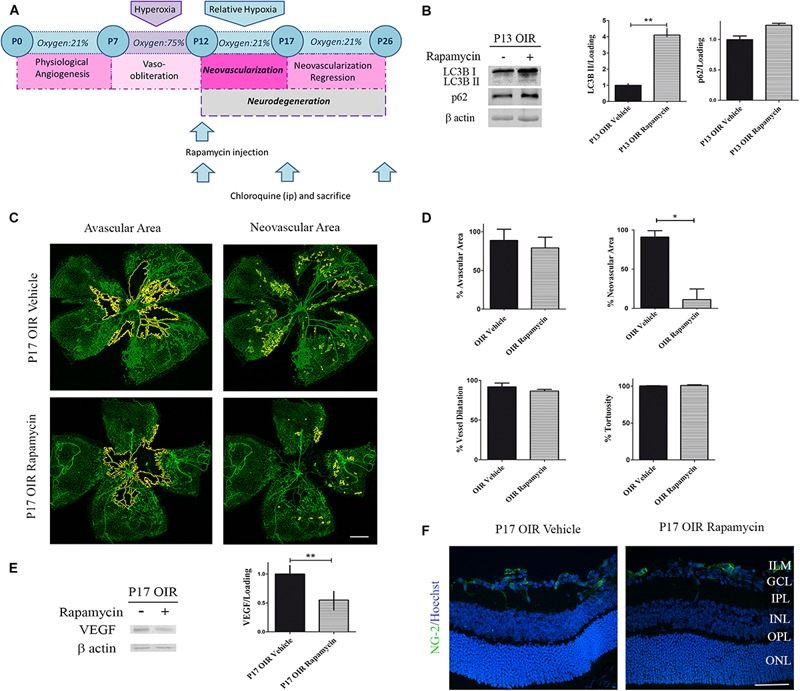
Effect of Rapamycin on the vascular alterations in the OIR mouse retinas. **(A)** Rapamycin treatment schedule. Rapamycin (0,5 μg/ml) or vehicle was intravitreally injected at P12 and evaluated at P13, P17, or P26 after 4 h of CQ administration. **(B)** Representative Western blot and quantification data of autophagy markers, LC3B II and p62, from neural retinal extracts of OIR mice intravitreally injected with vehicle or Rapamycin at P12 and evaluated at P13, 4 h after CQ administration. β-Actin is shown as a loading control. Graph shows results of three independent experiments. **(C)** Representative images of flat-mount retinas at P17 showing GSA-IB4 vascular staining in OIR-vehicle and OIR-Rapamycin mice. Areas with VO and NV are indicated. Scale bar: 500 μm. **(D)** The VO (%) was quantified as the ratio of central avascular area to whole retinal area, the NV (%) was quantified as a percentage of whole retinal area. In mayor vessels of the retina, diameter was quantified by tracing a transversal line to the vessel. The tortuosity was obtained by drawing a line along the vessel and comparing it to a straight line traced from the optic nerve to the first branching point. **(E)** Representative Western blot and quantification data of VEGF from neural retinal extracts in OIR mice intravitreally injected with vehicle or Rapamycin at P12 and evaluated at P17, 4 h after CQ administration. β-Actin is shown as a loading control. Graph shows results of three independent experiments. **(F)** Representative immunofluorescence analysis of NG-2 (green) in cryosections of OIR mice intravitreally injected with vehicle or Rapamycin at P12 and evaluated at P17, 4 h after CQ administration. Scale bar: 50 μm. ILM, inner limiting membrane; GCL, ganglion cell layer; IPL, inner plexiform layer; INL, inner nuclear layer; OPL, outer plexiform layer; ONL, outer nuclear layer. Data are presented as mean ± SEM. ^∗^*p* < 0.05, ^∗∗^*p* < 0.01.

Rapamycin is a potent inhibitor of the complex mTORC1. One of the functions of this multi-protein complex is to prevent the formation of new autophagosomes by phosphorylation of ATG13. In this sense, Rapamycin acts as an autophagy inductor and its different pharmacological formulations (everolimus, sirolimus, among others) have shown beneficial effects in cancer therapies (Li et al., 2014). On the other hand, mTORC1 phosphorylation increase VEGF synthesis, a trophic factor required for vessel survival, proliferation and migration (Wei et al., 2016). New insights in retinal neurodegeneration pointed Rapamycin as a promising therapy as it could both activate autophagy and decrease angiogenesis (Zhu and Du, 2018). Hence, we decided to evaluate neovascular and neurodegenerative processes in the OIR mouse model after Rapamycin treatment (Figure 3A). After a single intraocular injection at P12 OIR, we verified the increase in the autophagy flux 24 h later by Western blot assay (Figure 3B). In addition, as Yagasaki et al. (2014) demonstrated previously, Rapamycin decreased the NV in P17 OIR retinas. Quantitative analysis revealed a decrease of more than 75% of retinal NV area. However, the treatment was not able to attenuate other vascular alterations as avascular area, vessel dilatation and tortuosity (Figure 3C,D). In line with these observations, our next goal was to determine the protein expression levels of VEGF after Rapamycin treatment. Our results demonstrate that Rapamycin injection at P12 OIR significantly reduced VEGF protein expression in P17, respect to vehicle-injected OIR mouse samples (Figure 3E). Pericytes are mesenchymal cells that make important contributions to the microvascular tree in both normal and pathologic tissues (Gerhardt and Betsholtz, 2003). To determine the effect of Rapamycin on pericytes, retinal cryosections at P17 OIR were analyzed by immunofluorescence assay. Confocal microscopy analysis revealed no significant changes in NG-2 (a pericyte marker) staining after Rapamycin injection at P12 among both conditions (Figure 3F).

**FIGURE 4:**
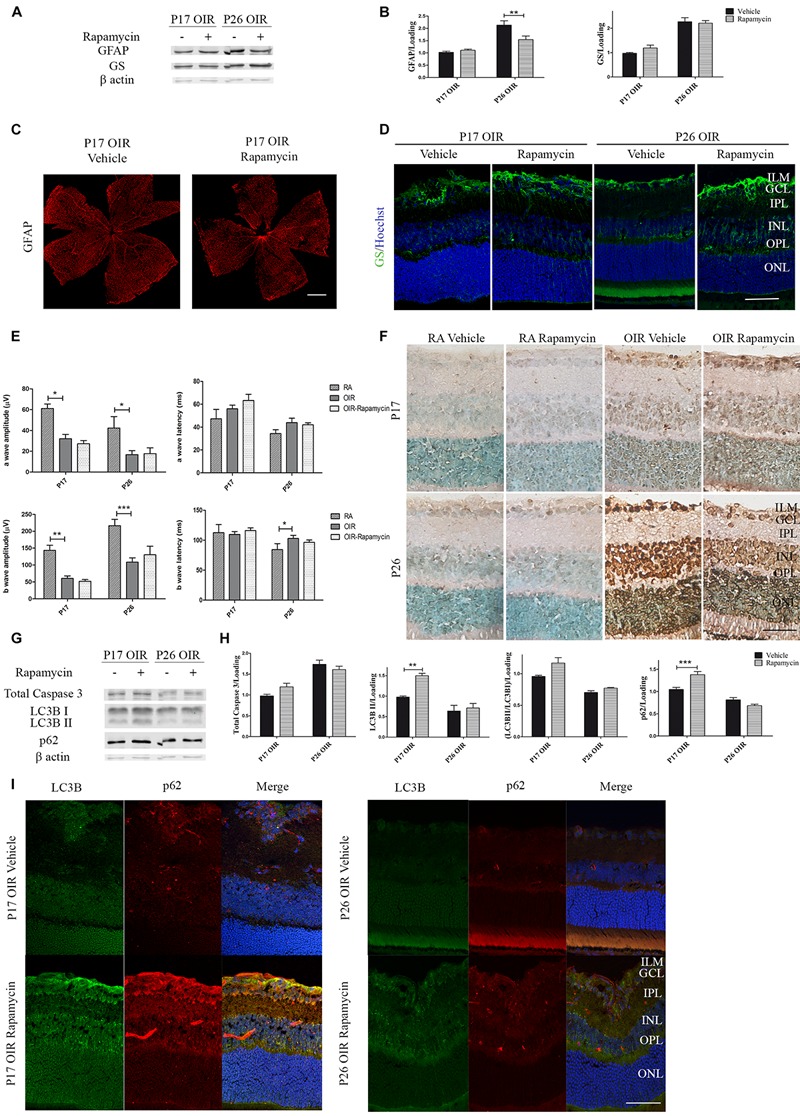
Effect of Rapamycin on the glial reactivity, neuronal functionality, and autophagy markers in OIR mouse retinas. **(A)** Representative Western blot of GS and GFAP from neural retina extracts of OIR mice intravitreally injected with vehicle or Rapamycin at P12 and evaluated at P17, 4 h after CQ administration. β-Actin is shown as a loading control. **(B)** Levels of GS and GFAP were quantified by densitometry and normalized to β-actin. Graph shows results of three independent experiments. **(C)** Representative images of flat-mount retinas at P17 showing GFAP staining in OIR-vehicle and OIR-Rapamycin mice. Scale bar: 500 μm. **(D)** Representative immunofluorescence analysis of GS (green) in cryosections of OIR mice intravitreally injected with vehicle or Rapamycin at P12 and evaluated at P17, 4 h after CQ administration. Scale bar: 50 μm. **(E)** Amplitudes and latencies of a- and b-waves from scotopic ERG were recorded at P17 and P26 in RA and OIR mice injected at P12 with vehicle or Rapamycin. Data show the average of responses of both eyes with eight mice per condition. **(F)** Representative TUNEL-labeled cryosections of RA and OIR mice injected with vehicle or Rapamycin at P12 and evaluated at P17 and P26. Scale bar: 50 μm. **(G,H)** Representative Western blot and quantification data of total caspase-3, LC3B II and p62 from neural retina samples of OIR mice intravitreally injected with vehicle or Rapamycin at P12 and evaluated at P17 and P26, 4 h after CQ administration. β-Actin is shown as a loading control. Graph shows results of three independent experiments. **(I)** Representative immunofluorescence analysis of LC3B (green) and p62 (red) in cryosections of OIR mice intravitreally injected with vehicle or Rapamycin at P12 and evaluated at P17 and P26, 4 h after CQ administration. Cell nuclei were counterstained with Hoechst 33258 (blue). Scale bar: 50 μm. ILM, inner limiting membrane; GCL, ganglion cell layer; IPL, inner plexiform layer; INL, inner nuclear layer; OPL, outer plexiform layer; ONL, outer nuclear layer. Data are presented as mean ± SEM. ^∗^*p* < 0.05, ^∗∗^*p* < 0.01, ^∗∗∗^*p* < 0.001.

Persistent glial activation is considered a bad prognosis sign, as macroglial reactive cells frequently secrete cytokines and chemokines, contributing to the proinflammatory environment and mediating neuronal death (Subirada et al., 2018). Recently, we have demonstrated that P17 OIR mouse retinas showed the highest neovascular profile and exhibited neuro-glial injury as well as retinal functional loss, which persisted until P26 OIR (Ridano et al., 2017). Herein, we further analyzed if the inhibition of mTORC1 improved gliosis. By Western blot assays we observed comparable protein expression of GFAP in Rapamycin and vehicle-injected P17 OIR retinas (Figure 4A,B), which was corroborated by confocal microscopy analysis of flat mounts retinas (Figure 4C). However, the quantification at P26 evidenced a significant decrease of the GFAP protein expression after Rapamycin injection at P12. At the same time, we evaluated glial ability to prevent excitotoxicity by analyzing GS protein expression. Quantification of protein levels of the detoxifying enzyme indicated that it was not modified after Rapamycin treatment neither at P17 nor at P26 (Figure 4A,B), although a slight increase in the inner layers was detected by immunofluorescence staining (Figure 4D).

In retinal neovascular pathologies there is a direct relationship between vascular changes and neuronal dysfunction (Kern, 2014). Thus, we performed ERG studies in both OIR groups (vehicle and Rapamycin) and recorded the intensity (amplitude) and the speed (latency) of the neuronal response after light stimuli. Results demonstrated that neurodegeneration associated to NV was not prevented by the injection at P12 of Rapamycin as ERG signals at P17 and P26 did not show differences after treatment (Figure 4E). In line with this result, TUNEL assay (Figure 4F) and Western blot quantification of total caspase-3, in retinal extracts of OIR mice (Figure 4G,H), were similar in Rapamycin-injected respect to vehicle-injected OIR mouse samples.

We also evaluated changes in autophagy flux at P17 and P26 OIR after Rapamycin treatment. As shown in Figure 4G,H, increased levels of LC3B II and p62 expression persisted up to P17. Immunofluorescence assays (Figure 4I), were consistent with the Western blot results demonstrating LC3B and p62 staining mainly at GCL and INL. Together, these results indicate that a single injection of Rapamycin was able to prevent the formation of neovessels at P17 OIR by decreasing VEGF protein expression and ameliorate gliosis at P26. By contrast, other vascular and neuronal abnormalities were not reverted.

### Anti-VEGF Treatment Increased the Autophagy Flux in OIR Mouse Retinas

**FIGURE 5:**
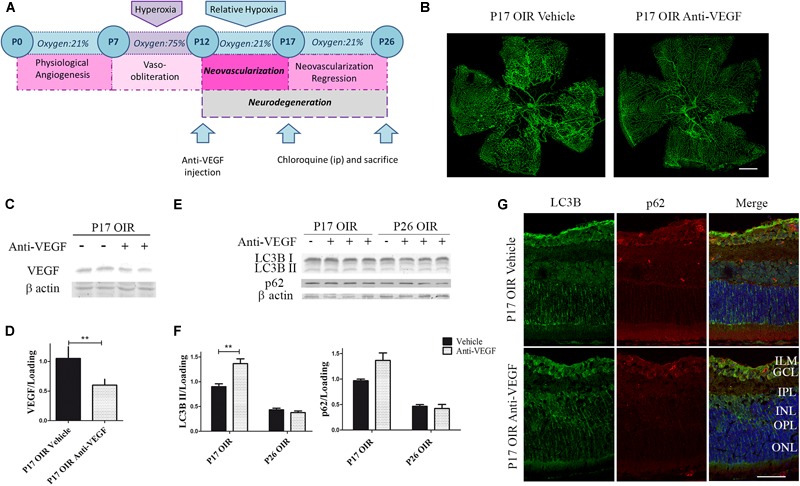
Effect of Anti-VEGF treatment on autophagy markers in the OIR mouse retinas. **(A)** Anti-VEGF mAb treatment schedule. Anti-VEGF mAb (1.25 μg) or vehicle was intravitreally injected at P12 and evaluated a P17 or P26, 4 h after CQ administration. **(B)** Representative images of flat-mount retinas at P17 OIR showing GSA-IB4 vascular staining in vehicle or anti-VEGF-injected eyes. Areas with VO and NV are indicated. Scale bar: 500 μm. **(C)** Representative Western blot of VEGF from P17 neural retinal extracts of OIR mice injected at P12 with vehicle or anti-VEGF mAb. β-Actin is shown as a loading control. **(D)** Levels of VEGF were quantified by densitometry and normalized to β-actin. Graph shows results of three independent experiments. **(E)** Representative Western blot of LC3B II and p62 from neural retina extracts of OIR mice intravitreally injected with vehicle or anti-VEGF mAb at P12 and evaluated at P17 and P26, 4 h after CQ administration. β-Actin is shown as a loading control. **(F)** Levels of LC3B II and p62 were quantified by densitometry and normalized to β-actin. Graph shows results of four independent experiments. **(G)** Representative immunofluorescence analysis of LC3B (green) and p62 (red) in cryosections of OIR mice intravitreally injected with vehicle or anti-VEGF mAb at P12 and evaluated at P17, 4 h after CQ administration. Cell nuclei were counterstained with Hoechst 33258 (blue). Scale bar: 50 μm. ILM, inner limiting membrane; GCL, ganglion cell layer; IPL, inner plexiform layer; INL, inner nuclear layer; OPL, outer plexiform layer; ONL, outer nuclear layer. Data are presented as mean ± SEM. ^∗∗^*p* < 0.01.

In recent years, several studies have shown that VEGF inhibitors can lead to the activation of autophagy, including ranibizumab, bevacizumab, and several other vascular inhibitors (Liang et al., 2015; Lytvynchuk et al., 2015; Liu et al., 2016). Thus, we decided to determine whether the classical anti-angiogenic treatment was able to modulate the autophagic flux. For this purpose, using a similar treatment schedule (Figure 5A), we verified the anti-VEGF effect on vascular alteration in flat-mount retinas, where a significant reduction in neovascular and avascular areas was observed (Figure 5B). In addition, as expected, the administration of anti-VEGF mAb at P12 significantly reduced VEGF protein expression in P17 respect to vehicle-injected OIR mouse samples (Figure 5C,D).

To determine the effect of anti-VEGF treatment on the autophagic process in the OIR mouse model we first examined the classical autophagy markers by Western blot assays. Quantitative analysis revealed a slight but significantly increase in LC3B II levels at P17 OIR (Figure 5E,F). This increase was corroborated by immunofluorescence assays where LC3B was detected mainly in GCL and INL (Figure 5G). These findings demonstrated that targeting VEGF prevented vascular alterations and also increased the autophagic flux in P17 OIR retinas.

### Spautin-1 Inhibited Autophagy, Reduced NV but Did Not Prevented Neurodegeneration in the OIR Mouse Model

**FIGURE 6:**
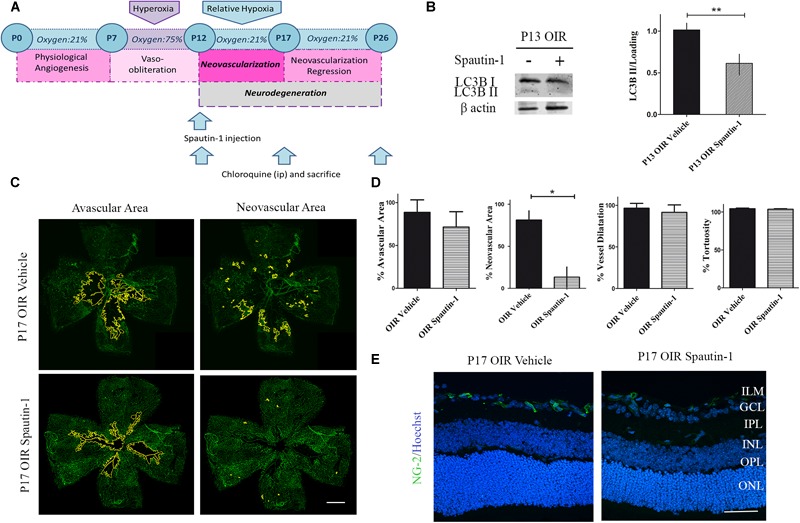
Effect of the Spautin-1 on the NV in OIR mouse retinas. **(A)** Spautin-1 treatment schedule. Spautin-1 (200 μM) or vehicle was intravitreally injected at P12 and evaluated at P17 or P26, 4 h after of CQ administration. **(B)** Representative Western blot and quantification data of autophagy marker LC3B II from neural retinal extracts of OIR mice intravitreally injected with Spautin-1 or vehicle at P12 and evaluated at P13, 4 h after CQ administration. β-Actin is shown as a loading control. Graph shows results of three independent experiments. **(C)** Representative images of flat-mount retinas at P17 showing GSA-IB4 vascular staining in OIR-vehicle and OIR-Spautin-1 mice. Areas with VO and NV are indicated. Scale bar: 500 μm. **(D)** The VO (%) was quantified as the ratio of central avascular area to whole retinal area, the NV (%) was quantified as a percentage of whole retinal area. In mayor vessels of the retina, diameter was quantified by tracing a transversal line to the vessel. The tortuosity was obtained by drawing a line along the vessel and comparing it to a straight line traced from the optic nerve to the first branching point. **(E)** Representative immunofluorescence analysis of NG-2 (green) in cryosections of OIR mice intravitreally injected with vehicle or Spautin-1 at P12 and evaluated at P17, 4 h after CQ administration. Scale bar: 50 μm. ILM, inner limiting membrane; GCL, ganglion cell layer; IPL, inner plexiform layer; INL, inner nuclear layer; OPL, outer plexiform layer; ONL, outer nuclear layer. Data are presented as mean ± SEM. ^∗^*p* < 0.05, ^∗∗^*p* < 0.01.

As autophagy seemed a key feature during NV, we next decided to decrease the autophagy flux by a single administration of Spautin-1 at P12 and to evaluate vascular, neuronal, and glial parameters at P17 and P26, respectively (Figure 6A). Western blot analysis showed a significant decrease in LC3B II expression at P13 (Figure 6B). Quantitative analysis of flat-mounts labeled with GSA-IB4 lectin showed a significant reduction of more than 70% in neovascular area after Spautin-1 treatment. However, other vascular alterations did not improved in OIR Spautin-1 mice, suggesting that autophagy is important for NV and that the activation in the OIR mouse is not mediating physiological revascularization (Figure 6C,D). In addition, a slight decrease in NG-2 staining was observed in Spautin-1 mice at P17 OIR (Figure 6E).

**FIGURE 7:**
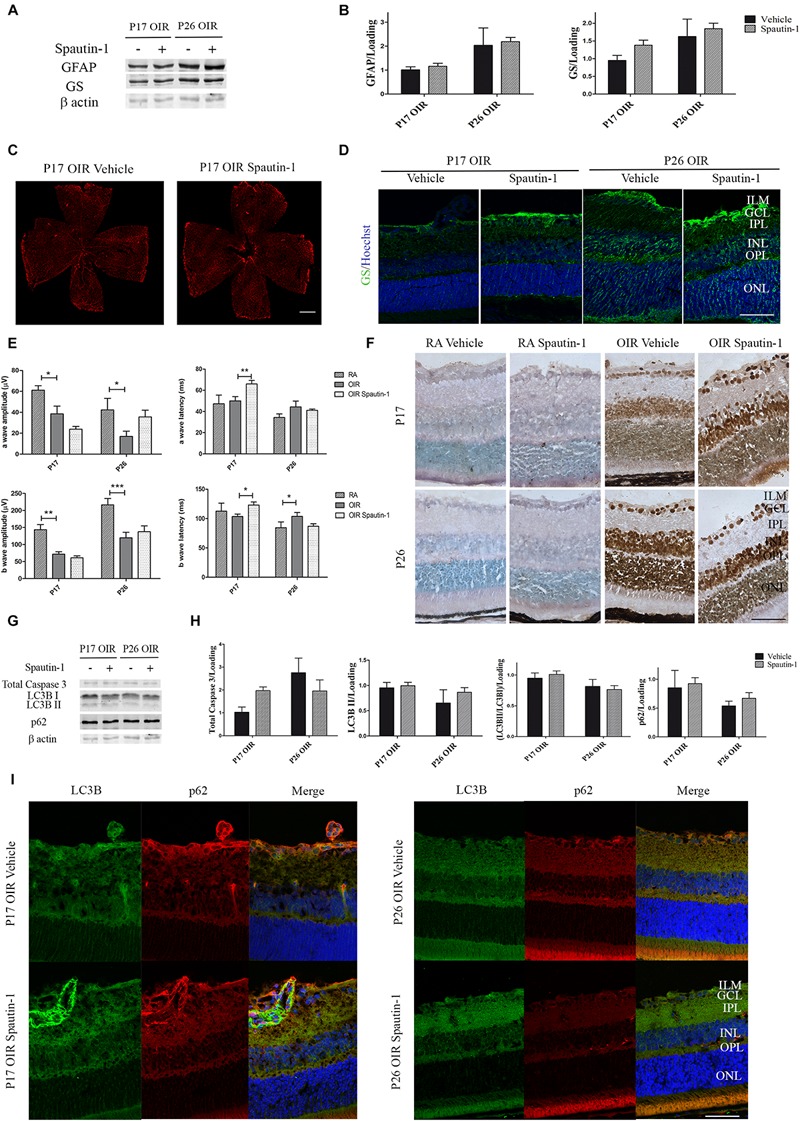
**(A)** Effect of Spautin-1 on the glial reactivity, neuronal functionality and autophagy markers in OIR mouse retinas. Representative Western blot of GFAP and GS from neural retina extracts of OIR mice intravitreally injected with Spautin-1 or vehicle at P12 and evaluated at P17, 4 h after CQ treatment. β-Actin is shown as a loading control. **(B)** Levels of GFAP and GS were quantified by densitometry and normalized to β-actin. Graph shows results of four independent experiments. **(C)** Representative images of flat-mount retinas at P17 showing GFAP staining in OIR-vehicle and OIR-Spautin-1 mice. Scale bar: 500 μm. **(D)** Representative immunofluorescence analysis of GS (green) in cryosections of OIR mice intravitreally injected with vehicle or Spautin-1 at P12 and evaluated at P17, 4 h after CQ administration. Scale bar: 50 μm. **(E)** Amplitudes and latencies of a- and b-waves from scotopic ERG were recorded at P17 and P26 in RA and OIR mice injected at P12 with vehicle or Spautin-1. Data show the average of responses in both eyes with eight mice per condition. **(F)** Representative TUNEL-labeled cryosections of RA and OIR mice injected with vehicle or Spautin-1 at P12 and evaluated at P17 and P26. Scale bar: 50 μm. **(G,H)** Representative Western blot and quantification data of total caspase-3, LC3B II and p62 from neural retina samples of OIR mice intravitreally injected with vehicle or Spautin-1 at P12 and evaluated at P17 and P26, 4 h after CQ administration. β-Actin is shown as a loading control. Graph shows results of three independent experiments. **(I)** Representative immunofluorescence analysis of LC3B (green) and p62 (red) in cryosections of OIR mice intravitreally injected with vehicle or Spautin-1 at P12 and evaluated at P17 and P26, 4 h after CQ administration. Cell nuclei were counterstained with Hoechst 33258 (blue). Scale bar: 50 μm. ILM, inner limiting membrane; GCL, ganglion cell layer; IPL, inner plexiform layer; INL, inner nuclear layer; OPL, outer plexiform layer; ONL, outer nuclear layer. Data are presented as mean ± SEM. ns, non-significant, ^∗^*p* < 0.05, ^∗∗^*p* < 0.01, ^∗∗∗^*p* < 0.001.

After Spautin-1 or vehicle treatment, no changes in GFAP levels were detected by Western blot of neural retinas samples (Figure 7A,B) or in flat-mount of retinas (Figure 7C). Similarly, no statistically differences in the expression of GS protein were found by Western blot (Figure 7A,B) or immunofluorescence (Figure 7D) after Spautin-1 treatment neither at P17 nor at P26.

In regard to neuronal functionality, the amplitude of the a-wave showed a tendency to decrease in OIR Spautin-1 mice at P17. In accordance, a later response (increased latency) was detected in photoreceptors as well as in inner neurons after autophagy inhibition (Figure 7E). Quantitative analysis of a-wave and b-wave amplitudes and latencies evidence no significant changes in neuronal functionality at P26. In line with these results, TUNEL staining showed a slight increase in the number of positive cells in the ONL after Spautin-1 treatment at P17 OIR (Figure 7F). Activation of apoptosis, measured by a reduction in total caspase 3 protein levels was not significantly different at both time points evaluated (Figure 7G,H). Finally, we evaluated protein expression of LC3B and p62 5 days (P17) or 14 days (P26) after Spautin-1 treatment. Quantitative analysis of Western blot assays (Figure 7G,H) and confocal microscopy images (Figure 7I) evidenced that both autophagy markers returned to OIR levels. These results showed that the inhibition of autophagy by Spautin-1 was harmful for the functionality and survival of photoreceptors in P17 OIR retinas.

## Discussion

Preretinal NV is a common feature in many diseases, including ROP, DR and Sickle cell retinopathy. Even though their pathophysiology is different, this family of retinopathies is characterized by the decrease in oxygen tension at a certain stage. The immediate response is the increase in VEGF synthesis, a trophic factor responsible of the proliferation of ECs that originate fragile and immature vascular tufts (Campochiaro, 2015; Rubio and Adamis, 2016). Simultaneously, other events take place in the retina as gliosis and neurodegeneration, which can be developed by the hypoxic stimulus or another primary injury (Ridano et al., 2017). Coincidentally, proliferative retinopathies treatments are palliative and aim to manage NV. However, ongoing inflammation, neuronal death, and impaired oxygen supply frequently lead to neovascular recurrence. Thus, previous studies indicated that a more appropriate therapy should encompass the normalization of both vascular and non-vascular alterations in proliferative retinopathies (Liang et al., 2012).

Modulation of autophagy flux results an attractive target, as this intracellular process occurs in every cell and has proven relationship with neurodegeneration, angiogenesis, and inflammation (Boya et al., 2016). Several research studies have demonstrated that inhibition or stimulation of the autophagic flux is able to reverse the alterations of multiple chronic or acute pathologies, including visual disturbances (Mitter et al., 2012; Russo et al., 2013; Chinskey et al., 2014; Frost et al., 2014; Chai et al., 2016; Rosa et al., 2016; Amato et al., 2018). Rapamycin, rapalogs and CQ are the most habitual choice for experimental design as they have been approved by FDA for a specific treatment (Nalbandian et al., 2015; Kezic et al., 2018) and it would be easier to execute clinical trials for second uses of these drugs.

Therefore, we initially explored changes in the autophagy flux in the OIR mouse model. To note, OIR model very closely recapitulates the pathologic events occurring in ROP and some aspects of proliferative DR (Smith et al., 1994). Treatments were carried out at different postnatal days when pups were still in developmental phase, where autophagy plays a crucial role (Boya et al., 2016). Indeed, Western blot assays in RA showed a decrease of LC3B II and p62 protein levels from P12 to P26, indicating a high autophagy flux early after birth. In contrast, OIR mice showed an increase in LC3B II and p62 protein levels at P17 OIR, compared to RA controls demonstrating a rise in autophagosomes in the cytosol of retinal cells. Transcriptional activity of autophagy proteins revealed a significant increase in p62 mRNA levels at P17OIR. Upon induction of the flux, p62 is degraded into the autophagosome and it would be necessary to synthesize more protein. This result would indicate that the increase in p62 protein levels at this time point is a consequence of the increased protein synthesis but not of its accumulation.

Physiological vascularization of the retina completes at P21 in mice, when the provisional hyaloid vasculature is replaced by the definite one following a hypoxic gradient. Firstly, the superficial plexus is formed by the radial sprouting of vessels from the optic nerve. Later, the deep plexus is formed by transversal outgrowth of superficial vessels and the retinal vasculature achieves completion with the formation of the intermediate plexus (Stahl et al., 2010). Due to the fact that there is an irregular oxygen supply in the different layers of the retina, the sensing of the hypoxic stimulus could vary along the neural retina and therefore the cellular response (Rodrigues et al., 2013). Confocal images showed that areas near the vascular plexus, GCs and neurons residing in the INL increased LC3B protein expression in P17 OIR mice, whereas autophagy markers were not modified in ONL neurons. Unchanged autophagic flux in photoreceptors could be explained by the provision of oxygen by the choroid vasculature to rods and cones, preventing severe hypoxia in this layer. Although photoreceptors are damaged in OIR model, a reduced cell death rate is observed (Ridano et al., 2017). Nearby to the ILM, both MGCs and ECs showed an increase in LC3B staining. Interestingly, at P17 OIR, LC3B-positive vesicles were found inside ECs in neovascular tufts, suggesting an increased autophagic flux in proliferating cells.

We further confirmed the activation in autophagy flux at P17 OIR by observing a greater number of LC3B/LAMP1 double labeled vesicles, indicating an increase in amphisomes in the inner layers of the retina compared to control mice. An increase in LAMP1 structures was observed after blockade of the flux with CQ, mainly in the ILM, evidencing that the degradation pathway is activated. In addition, neovessels in the ILM hydrolyzed DQ-BSA in lysosomes in great proportion. By TUNEL assay, we demonstrate that cell viability is reduced in INL and GCL. Indeed, neurons undergoing cell death or high hypoxic stress could activate autophagy flux as a survival mechanism.

Next we wonder if the administration of Rapamycin at P12 could improve vascular, glial, or neuronal alterations in OIR mice. We took into account that mTORC1 inhibition increases autophagy flux, but also modulates multiple intracellular pathways related to inflammation, angiogenesis and metabolism. A single intraocular injection of Rapamycin decreased the area occupied by neovessels, however the treatment did not promote physiological revascularization (evaluated by avascular area, vessel dilatation, and tortuosity). The normalization of the vascular plexus is essential for the proper irrigation of the retinal tissue and prevents future alterations derived from the turbulence in the blood flow or stasis of blood cells (Liang et al., 2012). The reduction in NV area was supported by the decrease in VEGF levels. It has been described that inhibition of mTORC1 decreases the synthesis of the transcription factor HIF-1α and consequently downregulates the expression of its target genes (Liu et al., 2015). Notably, in the retina VEGF is produced mainly in MGCs, astrocytes, GCs, retinal pigmented epithelium and ECs in a minor proportion (Wang et al., 2010). Then, it is possible that the modulation of the neovascular process is due to the direct effect of Rapamycin on other cells rather than endothelium. In fact, Rapamycin could be a more successful therapeutic strategy because it decreases the synthesis of VEGF, providing an adequate level of the trophic factor. The excessive inhibition of VEGF signaling would be detrimental for neurons. Under certain pathological conditions, increased autophagy flux can also induce cell death (Hirt et al., 2018). In our model, Rapamycin did not alter pericytes viability, one of the most susceptive cells in the vasculature. Persistence of mural cells is a good marker of vascular maturation, indicating that final stage of vasculogenesis is not altered and the newly formed vessels are functional.

Related to gliosis, Rapamycin markedly reduced GFAP expression at P26 OIR, indicating a decrease in the glial response during the neovascular regression stage. This would contribute to the reduction in the persistent pro-inflammatory response that promotes damage in the retinal tissue. Unfortunately, a single dose of Rapamycin was insufficient to increase neuronal functionality. Further studies would be necessary to evaluate if a prolonged administration schedule improves visual function.

Previously, it has been reported that high dose of Rapamycin increases the avascular area (Yagasaki et al., 2014) which could be a result of reduced proliferation or increased cell death of ECs. Here, the induction in autophagy flux did not resulted in an increase of apoptosis, measured by total caspase 3, suggesting that the crosstalk between both pathways is not the responsible of the caspase mediated cell death. Our results showed that the increase in autophagy flux continue up to P17 OIR, indicating that Rapamycin has a relatively long half-life in the vitreous, as previously reported (Nguyen et al., 2018).

In a comparative experiment, we analyzed the effect of the anti-VEGF therapy on the autophagy flux. Western blot analysis revealed a slight induction of the flux at P17 OIR. As confocal images showed an increase in the autophagy proteins, LC3B and p62, in GCL and INL, we consider that the deprivation of the trophic factor VEGF can activate survival mechanisms as autophagy. Previously, we reported that intravitreal administration of anti-VEGF does not improve neuronal functionality and gliosis (Ridano et al., 2017). Thereby, Rapamycin benefits over anti-VEGF therapy rely on the multiplicity of events modulated through the inhibition of mTORC1. Although both treatments reduced NV and activated autophagy, neither of them prevented neurodegeneration in our experimental conditions.

To unravel the role of autophagy in the OIR mouse model, we injected at P12 a single dose of Spautin-1, an inhibitor of the ubiquitin-specific peptidases USP10 and USP13, which target the Beclin-1 subunit of Vps34 complexes (Liu et al., 2011). Remarkably, vascular effects were similar to those observed with Rapamycin. Spautin-1 decreased the neovascular area without modifying other vascular parameters. In this sense, confocal images of neovessels shed light on this result demonstrating that proliferating ECs, highly metabolic cells, showed a fast autophagy flux. Thus, the inhibition of this process would lead to vascular death or a decreased proliferation rate. The interaction between ECs and pericytes is critical for the formation of a structurally sound microvasculature. In this study, we found a reduced NG-2 staining in OIR mice retinas after Spautin-1 treatment indicating an apparent decrease in pericyte density, which may exacerbate vascular alterations.

In regard to non-vascular cells, glial response remained unchanged as no modifications in GS and GFAP levels were detected after Spautin-1 treatment. Electroretinographic recording showed that photoreceptors functionality tend to decrease at P17 OIR in Spautin-1 treated mice. This result reinforces the idea that retinal layers with high autophagy flux at the neovascular peak activate this pathway as a survival mechanism. In this sense, the importance of the autophagic flow became more evident in the photoreceptors because it is the layer less affected in the OIR. Correlatively, an increase in TUNEL positive staining was observed in the ONL when autophagy was inhibited. Five days after Spautin-1 injection, LC3B II and p62 protein levels returned to OIR control levels, indicating a reduced half-life of the drug in the eye.

Presumably, all retinal cells are intended to activate autophagy flux in hypoxic conditions. However, the ability to respond will depend on the status of the cell as well as the intensity and duration of the stimuli (Boya et al., 2016). Prior to the hypoxic stage in the OIR model, the hyperoxic phase induces several alterations in retinal cells originated by apoptotic events at P12 (Sennlaub et al., 2002; Vecino et al., 2004; Narayanan et al., 2011). Recently, it has been demonstrated that retinas of OIR mice are characterized by increased apoptosis and decreased autophagy from P13 to P15 (Cammalleri et al., 2017). Here, using the same *in vivo* model, we completed the analysis including, in addition to P12 and P17, the vascular regression stage (P26). Overall, our results demonstrate that all treatments of induction or inhibition of the autophagic flux reduced neovascular area but were unable to completely reverse the neuronal damage. Besides, compared to current treatments, rapamycin provides a more promising therapeutic strategy as it reduces both neovascular tufts and persistent gliosis.

## Ethics Statement

C57BL/6J mice were handled according to guidelines of the ARVO Statement for the Use of Animals in Ophthalmic and Vision Research. Experimental procedures were designed and approved by the Institutional Animal Care and Use Committee (CICUAL) of the Faculty of Chemical Sciences, National University of Córdoba (Res. HCD 1216/18). All efforts were made to reduce the number of animals used.

## Author Contributions

PS and MS designed the experiments. PS, MP, MR, and VL conducted the experiments. PS, MP, MR, VL, and MS interpreted the data. GC and CF contributed new reagents, analytic tools, and analyzed and discussed the available data. PS and MS wrote and edited the manuscript.

## Conflict of Interest Statement

The authors declare that the research was conducted in the absence of any commercial or financial relationships that could be construed as a potential conflict of interest.

## Abbreviations

CQ: chloroquine
DR: diabetic retinopathy
ECs: endothelial cells
ERG: electroretinogram
GCL: ganglion cell layer
GFAP: glial fibrilar acid protein
GS: glutamine synthase
HIF: hypoxia inducible factor
ILM: inner limitant membrane
INL: inner nuclear layer
LAMP1: lysosomal-associated membrane protein 1
LC3B: microtubule-associated proteins 1A/1B light chain 3B
MGCs: Muller glial cells
NG-2: neuron-glial 2
NV: neovascularization
OIR: oxygen induced retinopathy
ONL: outer nuclear layer
P: post natal day
RA: room air
ROP: retinopathy of prematurity
VEGF: vascular endothelial growth factor
VO: vaso-obliteration
